# Schaaf-Yang syndrome shows a Prader-Willi syndrome-like phenotype during infancy

**DOI:** 10.1186/s13023-019-1249-4

**Published:** 2019-12-02

**Authors:** Yutaka Negishi, Daisuke Ieda, Ikumi Hori, Yasuyuki Nozaki, Takanori Yamagata, Hirofumi Komaki, Jun Tohyama, Keisuke Nagasaki, Hiroko Tada, Shinji Saitoh

**Affiliations:** 1 Department of Pediatrics and Neonatology, Nagoya City University Graduate School of Medical Sciences, Kawasumi 1, Mizuho-Cho, Mizuho-Ku, Nagoya, 467-8601 Japan; 2 Department of Pediatrics, Jichi Medical University, Tochigi, Japan; 3 Department of Child Neurology, National Center Hospital, National Center of Neurology and Psychiatry (NCNP), Tokyo, Japan; 4 Department of Child Neurology, Nishi-Niigata Chuo National Hospital, Niigata, Japan; 5 Division of Pediatrics, Niigata University Graduate School of Medical and Dental Sciences, Niigata, Japan; 6 Department of Pediatrics, Chibaken Saiseikai Narashino Hospital, Narashino, Japan

**Keywords:** *MAGEL2*, Schaaf-Yang syndrome, Genomic imprinting, Neurological deterioration

## Abstract

**Background:**

Schaaf-Yang syndrome (SYS) is a newly recognized imprinting related syndrome, which is caused by a truncating variant in maternally imprinted *MAGEL2* located in 15q11-q13*.* Yet, precise pathomechanism remains to be solved. We sequenced *MAGEL2* in patients suspected Prader-Willi syndrome (PWS) to delineate clinical presentation of SYS. We examined 105 patients with clinically suspected PWS but without a specific PWS genetic alteration. Sanger sequencing of the entire *MAGEL2* gene and methylation-specific restriction enzyme treatment to detect the parent of origin were performed. Clinical presentation was retrospectively assessed in detail.

**Results:**

Truncating variants in *MAGEL2* were detected in six patients (5.7%), including a pair of siblings. All truncating variants in affected patients were on the paternally derived chromosome, while the healthy father of the affected siblings inherited the variant from his mother. Patients with *MAGEL2* variants shared several features with PWS, such as neonatal hypotonia, poor suck, and obesity; however, there were also unique features, including arthrogryposis and a failure to acquire meaningful words. Additionally, an episode of neurological deterioration following febrile illness was confirmed in four of the six patients, which caused severe neurological sequalae.

**Conclusions:**

SYS can be present in infants suspected with PWS but some unique features, such as arthrogryposis, can help discriminate between the two syndromes. An episode of neurological deterioration following febrile illness should be recognized as an important complication.

## Background

Chromosome 15q11-q13 contains a cluster of imprinted genes essential for normal mammalian neurodevelopment [1]. Paternal deletions of this region result in Prader-Willi syndrome (PWS, OMIM #176270) [1, 2]. It was recently reported that truncating variants in *MAGEL2*, one of the paternally expressed genes located in this region, caused Schaaf-Yang syndrome (SYS, OMIM #615547) [3–10]. SYS patients present with several symptoms typical of PWS, such as developmental delay, neonatal hypotonia, poor suck that requires special feeding techniques, and excessive weight gain. However, they also experience symptoms not typically seen in PWS, including arthrogryposis and autism spectrum disorder (ASD) [3–10]. Truncating variants in *MAGEL2* cause severe arthrogryposis with reduced fetal movement resulting in perinatal death [11]. Recently, pathogenic variants in *MAGEL2* were reported as causes of Chitayat-Hall syndrome, which is characterized by distal arthrogryposis, intellectual disability, dysmorphic features and hypopituitarism, and in particular, growth hormone deficiency (OMIM #208080) [12–14]. These reports demonstrated that SYS and Chitayat-Hall syndromes are allelic disorders.

The pathological mechanism underlying SYS is thought to be the loss of functional *MAGEL2* [3, 5], yet a gain-of-function mechanism has also been suggested [9, 15]. *MAGEL2* is located in the PWS critical region and is deleted in deletion-positive PWS patients. Therefore, a loss-of-function phenotype in *MAGEL2* should also be associated with PWS. However, the paradox is that patients with SYS show more severe phenotypes than deletion-positive PWS patients. Interestingly, deletions of the entire paternal copy of *MAGEL2* can cause different phenotypes like other conditions with a mild phenotype caused by a segmental deletion including other genes [16, 17].

In this study, we sequenced 105 patients with clinically suspected PWS but without a genetic alteration specific for PWS, and examined their clinical features.

## Methods

### Study subjects

The subjects were 105 patients in whom PWS was clinically suspected but not genetically diagnosed. We initially performed the *SNURF-SNRPN* and *MEG3* DNA methylation tests. The *MEG3* methylation test was performed to detect Temple syndrome, which demonstrates a PWS-like phenotype [18]. Subjects who tested negative in both tests were enrolled in the study. Experimental protocols were approved by the Ethical Committee for the Study of Human Gene Analysis at Nagoya City University Graduate School of Medical Sciences, and were carried out in accordance with the approved guidelines. Written informed consent was obtained from the parents of patients.

### Genetic analyses

The *SNURF-SNRPN* and *MEG3* DNA methylation tests were performed as described previously [18, 19]. Long-range PCR of the *MAGEL2* coding region was performed as previously reported [3], followed by Sanger sequencing using a 3130xl DNA Analyzer (Thermo Fisher Scientific, Waltham, MA, USA) with six sequencing primer pairs (primer sequences are available on request). If a variant was detected, the parental origin of the variant was determined using the methylation-specific restriction enzyme, SmaI (New England Biolabs, Beverly, MA, USA), as previously reported [3].

## Results

### Molecular genetics

**Fig. 1:**
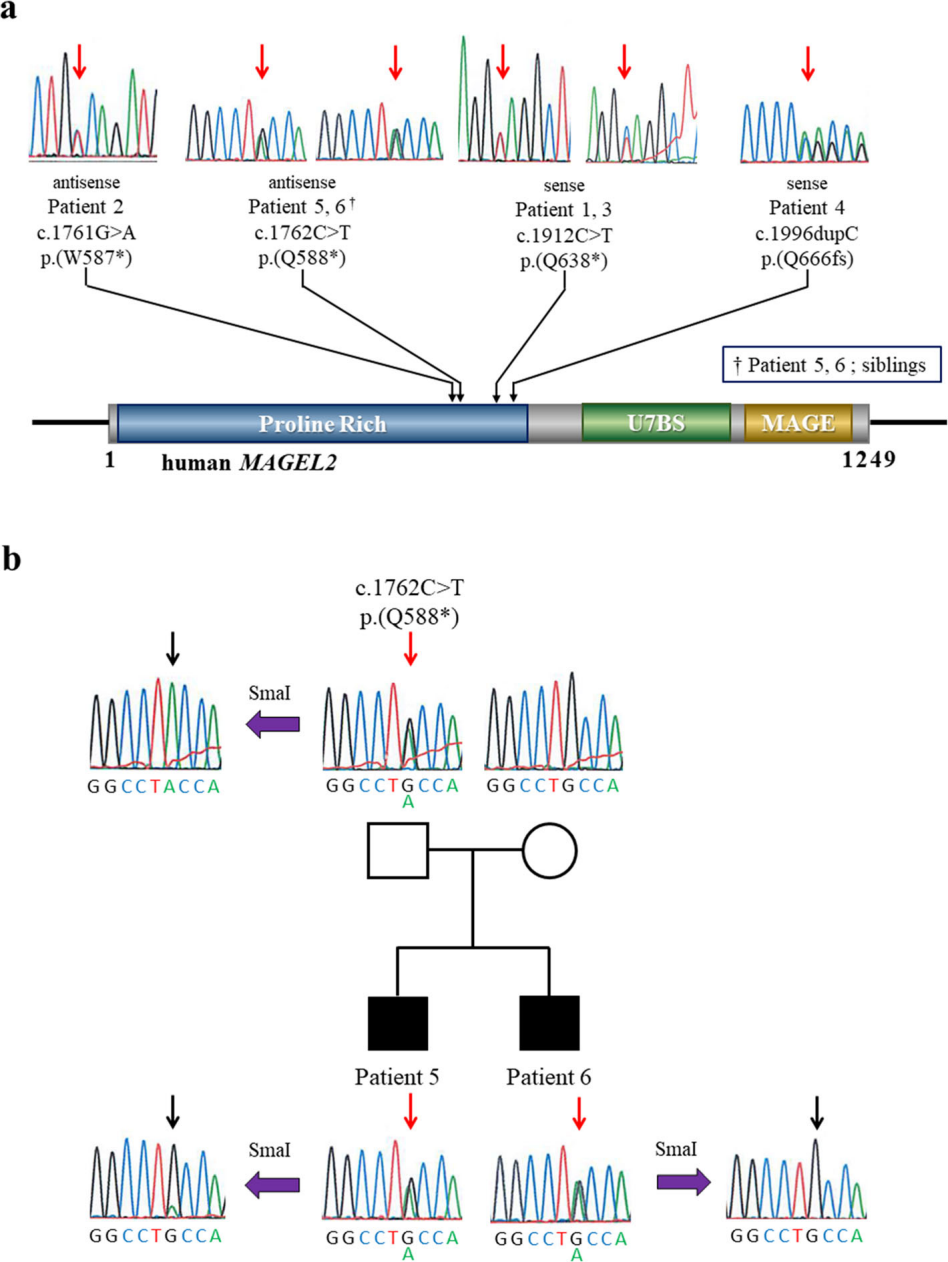
**a** *MAGEL2* variants and protein domain structures. Truncating *MAGEL2* variants found in our cohort and previously reported in literature (RefSeq NM_019066.4). The six variants identified in our patients with Sanger sequencing confirmation are shown above the gene. The proline-rich region (Proline Rich; residues 13–700), USP7 binding site (U7BS; residues 949–1004), and MAGE homology domain (MAGE; residues 1020–1219) are indicated by their positions in the coding sequence. **b** Pedigree of a familial case and phasing of the *MAGEL2* variant. The c.1762C > T variant identified in patients 5 and 6, who were siblings, was found to be inherited from the unaffected father and unaffected paternal grandmother. Sanger sequencing following SmaI digestion detected only the methylated allele (maternal allele). Red arrows indicate the *MAGEL2* c.1762C > T variant site. Black arrows indicate the variant site after SmaI digestion

Among 105 patients with clinically suspected PWS but no PWS-specific genetic alteration, we identified truncating variants in *MAGEL2* in six patients (five nonsense variants, one frameshift variant), including a pair of siblings (Fig. 1a). The variants were all centrally located within the single exon gene in amino acids 587 to 666. All truncating *MAGEL2* variants were on the paternal allele. Additionally, 25 nonsynonymous single nucleotide variants were also identified (Table 1). The latter are likely to have no pathologic significance as they are rare SNPs registered in public databases, including the Human Genetic Variation Database (HGVD; http://www.hgvd.genome.med.kyoto-u.ac.jp) [20] and Exome Aggregation Consortium (EXaC; http://exac.broadinstitute.org), or they are maternally inherited.

**Table 1:** Nonsynonymous SNVs identified in our study

Genomic description (GRCh38, NC_000015.10)	mRNA (NM_019066.4)	Protein	MAF in our study	MAF in HGVD	dbSNP151	Inheritance
g.23645492 T > C	c.2251A > G	p.(I751V)	2/210 (0.009)	0.037	rs374443204	NA
g.23645941G > T	c.1802C > A	p.(P601Q)	1/210 (0.005)	0	No	Maternal
g.23645974G > T	c.1769C > A	p.(P590H)	1/210 (0.005)	0.004	No	NA
g.23646356C > G	c.1387G > C	p.(A463P)	5/210 (0.02)	0.034	rs2233063	NA
g.23646383C > T	c.1360G > A	p.(A454T)	1/210 (0.005)	0.011	rs548001629	NA
g.23646439G > A	c.1304C > T	p.(P435L)	3/210 (0.01)	0.007	rs2233062	NA
g.23646644C > A	c.1099G > T	p.(G367C)	2/210 (0.01)	0.002	rs576068111	NA
g.23646905 T > G	c.838A > C	p.(K280Q)	1/210 (0.005)	0.008	rs1177517513	NA
g.23646890C > T	c.853G > A	p.(G285R)	5/210 (0.025)	0.024	rs143908070	NA
g.23647282G > C	c.461C > G	p.(P154R)	1/210 (0.005)	0.001	rs1460068607	NA
g.23647346A > G	c.397 T > C	p.(S133P)	1/210 (0.005)	0.002	rs1410473331	NA
g.23647424C > T	c.319G > A	p.(V107I)	1/210 (0.005)	0.003	rs922355971	NA
g.23647456C > T	c.287G > A	p.(G96E)	1/210 (0.005)	0	No	Maternal

*HGVD* Human Genetic Variation Database, *MAF* minor allele frequency, *NA* not available

The c.1762C > T; p (Q588*) variant identified in the siblings (patients 5 and 6) was found to be inherited from the unaffected father, where the variant of the father was on his maternal allele (Fig. 1b). The remaining three variants in patients for whom both parent’s DNA were available for analysis were confirmed to be de novo*.* Parent samples from Patient 1 were not available.

### Clinical features

**Fig. 2:**
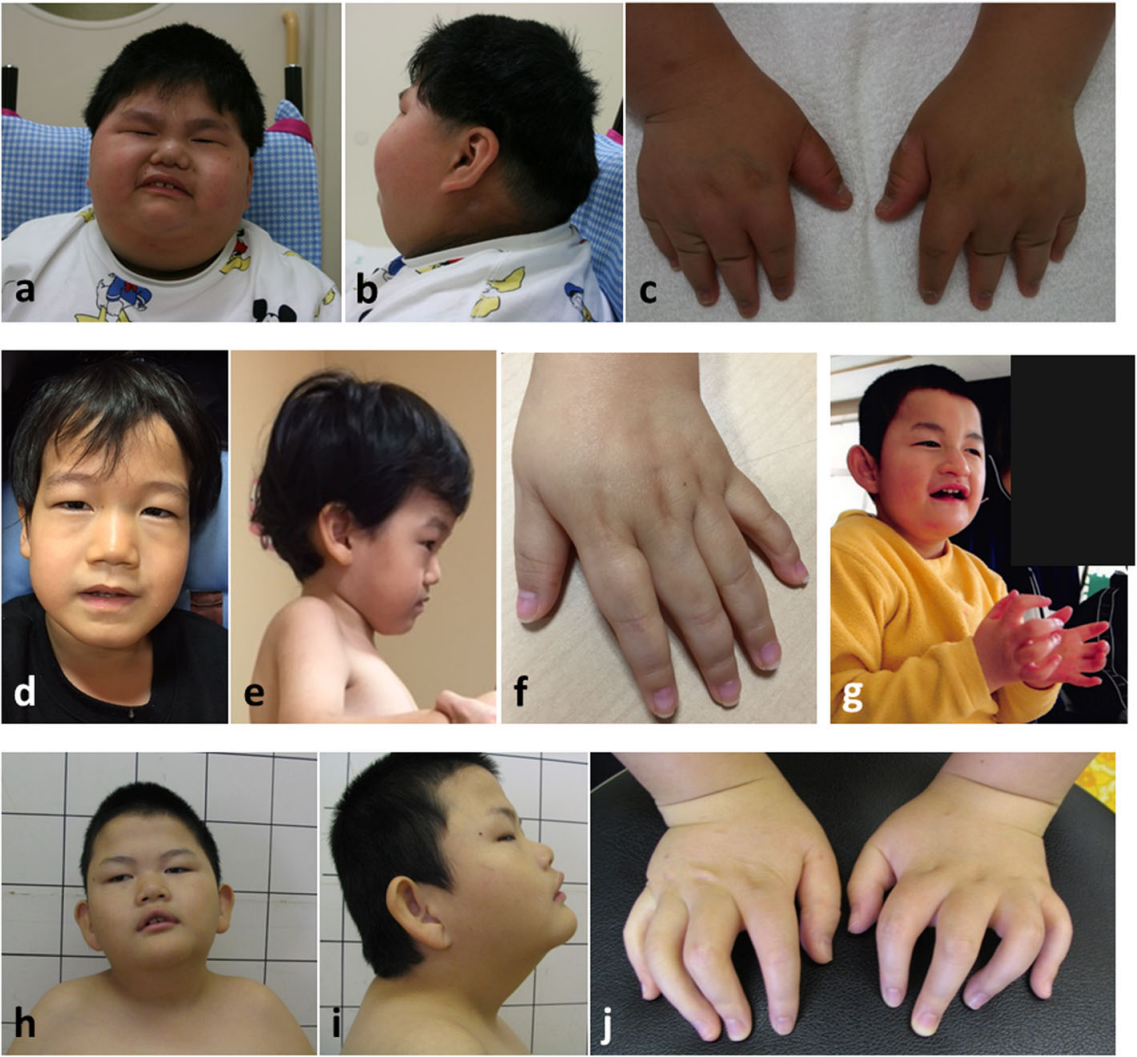
Clinical photographs of patient 2 (**a**, **b**, **c**), patient 4 (**d**, **e**, **f**), patient 5 (**h**, **i**, **j**) and patient 6 (**g**). Most patients show a shallow nasolabial fold, large ears, and contractures of the proximal and distal interphalangeal joints, small hands, and tapering of the fingers

Table 2 summarizes the clinical features of the six patients harboring truncating variants in *MAGEL2*. Neonatal hypotonia, poor suck, and developmental delay, all major symptoms of PWS, were confirmed in all subjects. Typical clinical features are illustrated in Fig. 2. Characteristic facial appearance, and hypogonadism, also major symptoms of PWS, were confirmed in the majority of patients. Short stature and small hands, minor criteria of PWS, were also confirmed in 5/6 and 6/6 patients, respectively. All patients showed arthrogryposis, which is not typically seen in PWS. In addition, growth hormone (GH) deficiency, seen in Chitayat-Hall syndrome, was observed in all three patients in whom GH was evaluated. The developmental quotient, measured in only three patients, was 13–21, indicating severe intellectual disability. Interestingly, an episode of neurological deterioration following febrile illness was confirmed in four of the six patients. Patient 2 developed a seizure and impaired consciousness on the second day after the onset of fever. Brain MRI revealed high signal intensity areas in the right parietal white matter on diffusion-weighted imaging. He became unable to walk because of neurological sequelae. Patient 6 developed vomiting and a cluster of seizures on the third day after the onset of fever and was placed on a mechanical ventilator. Brain MRI revealed high signal intensity areas in bilateral putamen and globus pallidus on T2-weighted imaging. He became bedridden after an episode of acute encephalopathy.

**Table 2:** Clinical features of patients with a *MAGEL2* variant

	Patient 1	Patient 2	Patient 3	Patient 4	Patient 5	Patient 6
Sex	F	M	M	M	M	M
Variant	c.1912C > T	c.1761G > A	c.1912C > T	c.1996dupC	c.1762C > T	c.1762C > T
	p.(Q638*)	p.(W587*)	p.(Q638*)	p.(Q666fs)	p.(Q588*)	p.(Q588*)
Inheritance	not maternal	de novo	de novo	de novo	paternal	paternal
Affected allele	paternal	paternal	paternal	paternal	paternal	paternal
Gestational age	39w3d	42w0d	37w4d	38w2d	38w3d	39w0d
Birth weight, g (SD)	2400 (−1.6)	3265 (0.7)	2315 (−1.3)	2925 (0.2)	2980 (0.3)	2906 (−0.5)
Birth length, cm (SD)	46 (−1.6)	48 (−0.6)	NA	47.5 (−0.4)	48 (−0.2)	48 (−0.4)
Birth OFC, cm (SD)	34 (0.6)	32 (−1.0)	NA	34.5 (1.1)	33.8 (0.5)	35.2 (1.5)
Last follow up age	5y0m	3y6m	1y0 m	6y3m	23y5m	12y0m
Last weight, kg (SD)	20 (1.1)	18 (1.8)	4.2 (−5.7)	14 (−2.0)	72 (0.9)	24 (−1.9)
Last length, cm (SD)	90 (−3.9)	NA	NA	98 (−3.5)	162 (−1.5)	120 (−3.8)
BMI (Z-Score)	24.7 (2.85)	NA	NA	14.6 (−0.71)	27.4 (1.11)	16.7 (− 0.55)
PWS major criteria						
Neonatal hypotonia, poor suck	+	+	+	+	+	+
Feeding problems in infancy, with need for special feeding technique	+	–	+	+	+	–
Excessive weight gain before age 6 years	+	+	NA	–	+	–
Characteristic facial features	+	+	–	+	+	–
Hypogonadism	–	+	+	–	+	+
Developmental delay	+	+	+	+	+	+
PWS minor criteria						
Infantile lethargy, weak cry	+	–	+	+	+	+
Short stature	+	+	NA	+	+	+
Characteristic behavior (temper tantrums, violet outbursts, oppositional behavior, etc.)	–	–	NA	–	+	–
Hypopigmentation	–	+	+	–	+	+
Small hands	+	+	+	+	+	+
Eye abnormalities	+	–	+	+	+	+
Skin picking	–	+	–	+	+	+
Sleep apnea	–	–	NA	–	+	–
Other						
DQ (assessed method) age	13 (Enjoji) 5y	DD	NA	21 (K-test) 6y	21 (Enjoji) 12y	DD
Autism spectrum disorder	NA	NA	NA	NA	+	NA
Neurological deterioration	+	+	+	–	–	+
Arthrogryposis	+	+	+	+	+	+
GH deficiency	+	NA	NA	+	+	NA

*DD* apparently developmentally delayed but not scored by a standardized method, *DQ* developmental quotient, *Enjoji* Enjoji Scale of Infant Analytical Development, *F* female, *GH* growth hormone, *K-test* the revised version of Kyoto Scale of Psychological Development, *M* male, *NA* not available, *OFC* occipitofrontal circumference, *SD* standard deviation. Symbols: +, present; −, absent

*nonsense variant

## Discussion

In this study, we identified *MAGEL2* truncating variants in six of 105 (5.7%) patients initially suspected to have PWS, but excluded following specific genetic testing. These variants, including two novel variants, were centrally localized to a previously reported hotspot at amino acids 587–666 [5, 9]. To clarify how SYS and PWS share clinical features, we examined the clinical presentation of our patients with SYS. The major symptoms overlapping of PWS were hypotonia, poor sucking and developmental delay confirmed in all patients. We also found that four of the six (66.7%) patients in our cohort showed hypopigmentation, which is commonly seen in PWS but not previously reported in SYS. Hypopigmentation is thought to be caused by a dysfunction of *OCA2* or *GABRB3* [21], not by *MAGEL2*, and thus it was not expected. Yet, since our cohort was initially suspected of having PWS, an inclusion bias may be present. It was recently reported that Chitayat-Hall syndrome was also caused by pathogenic variants in *MAGEL2* [12, 14]. GH deficiency, which is a characteristic of this syndrome, was found in all three cases in whom GH was evaluated. Therefore, GH deficiency should also be assessed in SYS. Interestingly, in a recent paper, Patak et al. produced a review of their cases and a systematic review and found no clinical or genetic differences between SYS and Chitayat–Hall syndrome [14]. In addition, arthrogryposis, which is not found in PWS, was confirmed in all patients as previously reported [3, 5–10]. Many of the intellectual disabilities confirmed in this cohort were severe. No patients acquired meaningful words. The mean intelligence quotient (IQ) of PWS patients is 60–70 [22], therefore intellectual disability in SYS patients is significantly more severe than PWS patients. However, Patients 1, 2, 3 and 6 may have been affected by neurological deterioration due to encephalopathy-like episodes. Interestingly, four of the six patients had an episode of neurological deterioration following febrile illness. Although McCarthy et al. reported that 67% of patients with SYS showed temperature instability [9], no episodes of neurological deterioration have been reported. In Japan, the prevalence of febrile seizures and acute encephalopathy is high [23, 24], which may be associated with the high prevalence of encephalopathy-like episodes in this population. In addition, because post-febrile regression is difficult to identify, particularly in patients with severe intellectual disabilities, some patients may have been overlooked. Therefore, careful observation should be performed for patients with SYS during febrile illness. Although it was reported that SYS patients have a higher prevalence of ASD [3, 5, 9], this could not be evaluated due to the severe intellectual disability in most of our patients. Previous studies report that among adolescent patients with SYS, the proportion of those who presented with hyperphagia and obesity was low, unlike that of patients with PWS, and many of those presented with ASD [9, 14]. In our study, it was difficult to evaluate patients in these respects because we could only observe two patients until puberty; however, we identified some patients without hyperphagia, obesity, or the personality characteristic of PWS. Overall, our study finds that symptoms of SYS are distinct from, and more severe than, those of PWS.

Next, a genotype–phenotype correlation is discussed. Previous reports have mentioned that c.1996dupC is the most common variant, which is more severe than other variants (e.g., frequencies of arthrogryposis, tube feeding, and respiratory dysfunction) [9, 14]. In our study, however, Patient 4 (c.1996dupC) had a history of tube feeding during infancy, but did not show respiratory dysfunction, indicating that the patient’s condition was comparable to that of other patients at least by the age of 6 years.

Buiting et al. [17] reported a 3-year-old boy with a paternally inherited approximately 3.9 Mb deletion that spanned *MAGEL2* but not the *SNORD116* cluster. The patient showed mild motor delay, which appears to be different to the SYS phenotype. Therefore, the truncated variants of *MAGEL2* identified in SYS may cause different phenotypes to a complete deletion of *MAGEL2*, which is likely to represent loss of function. However, it should be noted that the 3.9 Mb deletion may not represent loss of function of *MAGEL2* because it could disturb proper expression of some other genes. This can only be established however, once more cases with a complete deletion of *MAGEL2* are reported. It should also be noted that almost only truncated variants but only one missense variant have been identified in *MAGEL2* [14]. If loss of function is the major pathomechanism, a missense variant, especially at a functional domain, would be expected. As *MAGEL2* is a single exon gene, nonsense-mediated mRNA decay is not induced. Therefore, truncating variants in *MAGEL2* may result in abnormal truncated protein products through a gain-of-function mechanism. Although several articles have raised the possibility of a dominant negative mechanism [5, 15], it seems that a gain-of-function mechanism is the most likely pathological mechanism because there is no proof that MAGEL2 forms multimers.

MAGEL2 belongs to the MAGE family proteins that were initially identified as tumor-specific antigens [25]. Proteins encoded by the MAGE gene family, with approximately 40 unique members in humans, share the MAGE homology domain that mediates protein-protein interaction [25, 26]. MAGEL2 is known to bind and to enhance the activity of the TRIM27 E3 RING ubiquitin ligase. The MAGEL2-USP7-TRIM27 (MUST) complex plays an important role in a cellular process that recycles membrane proteins from endosomes through the retromer sorting pathway [15, 27, 28]. Thus, dysregulation of this pathway may be associated with the pathogenesis of SYS. Indeed, retromer has been implicated in several neurodegenerative disorders in humans, including Alzheimer’s and Parkinson’s disease [29, 30].

## Conclusions

We identified truncating variants in *MAGEL2* in six of 105 patients with suspected PWS. SYS can be present in infants suspected of PWS, but some unique features, such as arthrogryposis, can help discriminate between the two syndromes. An episode of neurological deterioration following febrile illness should be recognized as an important complication.

## Data Availability

The datasets supporting the conclusions of this article are available in the ClinVar repository (http://www.ncbi.nlm.nih.gov/clinvar/).

## Abbreviations

ASD: Autism spectrum disorder
GH: Growth hormone
IQ: Intelligence quotient
PCR: Polymerase chain reaction
PWS: Prader-Willi syndrome
SNP: Single nucleotide polymorphism
SYS: Schaaf-Yang syndrome
